# Provenance-specific responses to climatic mismatch in *Betula ermanii* Cham. and implications for climate adaptation

**DOI:** 10.7717/peerj.21425

**Published:** 2026-06-11

**Authors:** Aye Myat Myat Paing, Takaki Aihara, Yoshihiko Tsumura, Mitsuru Hirota, Toshiya Yoshida, Kosuke Homma, Hajime Kobayashi, Atsuhiro Iio, Nobuhiro Tomaru, Dai Nagamatsu, Masahiro Takagi, Ikutaro Tsuyama, Yoko Hisamoto, Haruhiko Taneda, Susumu Goto

**Affiliations:** 1 Graduate School of Agricultural and Life Sciences, The University of Tokyo, Tokyo, Japan; 2 Graduate School of Life and Environmental Sciences, University of Tsukuba, Tsukuba, Japan; 3 Faculty of Life and Environmental Sciences, University of Tsukuba, Tsukuba, Japan; 4 Field Science Center for Northern Biosphere, Hokkaido University, Nayoro, Japan; 5 Sado Island Center for Ecological Sustainability, Niigata University, Sado, Japan; 6 Faculty of Agriculture, Shinshu University, Kamiina-gun, Japan; 7 Graduate School of Integrated Science and Technology, Shizuoka University, Shizuoka, Japan; 8 Graduate School of Bioagricultural Sciences, Nagoya University, Nagoya, Japan; 9 Faculty of Agriculture, Tottori University, Tottori, Japan; 10 Faculty of Agriculture, University of Miyazaki, Miyazaki, Japan; 11 Hokkaido Research Center, Forestry and Forest Products Research Institute, Sapporo, Japan; 12 Nikko Botanical Gardens, Graduate School of Science, The University of Tokyo, Tokyo, Japan

**Keywords:** Climate change, *Betula ermanii* Cham., Provenance trial, Assisted migration, Climate refugia, Boreal forest

## Abstract

Climate change is expected to alter the growth and survival of forest trees, but the magnitude and direction of these effects remain uncertain at the intraspecific level, particularly across provenances adapted to contrasting climatic conditions. For *Betula ermanii* Cham., a dominant deciduous tree in cool-temperate and subalpine forests of Japan, it remains unclear how climatic mismatches between provenance origin and planting environment influence early-life performance. Here, we quantified the effects of climatic transfer distances on seedling performance using data from 11 provenance trials distributed across Japan. Performance was defined as a composite index integrating survival and growth (mean height × survival rate) at the provenance × site level. Generalized linear mixed models were used to evaluate the effects of temperature and precipitation mismatches, and the fitted models were applied to future climate scenarios based on Shared Socioeconomic Pathways (SSPs), specifically SSP2–4.5 and SSP5–8.5, to project changes in performance. Seedling performance showed a weak decline with increasing precipitation at the planting site relative to the provenance origin, while temperature responses varied substantially among provenances. This heterogeneity indicates strong genotype-by-environment interactions, with high-elevation central provenances showing greater sensitivity to warming, whereas northern provenances were comparatively less responsive. Under future climate scenarios, northern provenances generally maintained higher predicted performance *in situ*, while central provenances showed reduced performance at their origins but improved performance when projected into northern regions, suggesting potential for assisted migration. In contrast, the southern rear-edge provenance exhibited consistently low performance across scenarios, indicating limited adaptive potential under climatic change. Overall, our results demonstrate that responses to climatic mismatch vary among provenances, and that incorporating provenance-specific climatic sensitivity improves predictions of future performance. These findings emphasize the importance of provenance-based strategies for forest management, including the identification of climate-resilient source provenances, evaluation of assisted migration options, and conservation of genetically distinct but vulnerable provenances.

## Introduction

Climate change has a profound effect on forest growth and mortality (Girardin et al., 2016; Taylor et al., 2017). Although some forest trees may benefit from improved growth conditions (McKenney et al., 2007), shifts in temperature and precipitation patterns can lead to enhanced and reduced growth, as highlighted in earlier research works (Boisvenue & Running, 2006; Wang et al., 2012). In particular, boreal regions are projected to experience warming by as much as 10 °C, whereas tropical areas may experience temperature increases of around 3 °C–4 °C by the century’s end (Masson-Delmotte et al., 2022). The plausible future climatic conditions for boreal regions include higher temperatures, longer growing seasons, and more frequent climate extremes, such as prolonged drought by the end of the century (Allan et al., 2021; Price et al., 2013; Stocker et al., 2013). Considering that boreal forests contain approximately 30% of the total carbon sequestered by the world’s forests (Pan et al., 2011), changes in boreal forest productivity markedly influence global climate systems (Ma et al., 2012). Therefore, understanding the impacts of climate change on boreal forest productivity is essential for mitigating such impacts.

Heating and provenance trials mimicking climate warming have revealed that the impacts of climate change differ among forest tree species (Dusenge, Madhavji & Way, 2020; Song et al., 2021; Sáenz-Romero et al., 2019). In a 5-year open-air experiment conducted on nine tree species (four evergreen conifers and five deciduous broad-leaved tree species), warming treatments of +1.6 °C and +3.1 °C were observed to increase their juvenile mortality (Reich et al., 2022). However, at +3.1 °C, conifers generally exhibited more negative responses, whereas broad-leaved species showed comparatively positive growth responses. These findings indicate that deciduous broad-leaved species may be relatively more tolerant to elevated temperatures than boreal conifers, although warming increases mortality overall.

Intraspecific variation, that is, genetic and phenotypic differences among provenances within a species, plays a critical role in shaping the mechanism by which forests respond to current and future climate changes (Leites & Benito Garzón, 2023; Rehfeldt et al., 2002; Reich & Oleksyn, 2008). Local adaptation occurs when individuals from a provenance perform better in their home environment than those from other provenances of the same species (Kawecki & Ebert, 2004). Considering that environmental conditions are often harsher or more variable at the margins of a species’ range, stronger selection pressures can lead to more pronounced local adaptation in marginal provenances compared with central provenances (Reich & Oleksyn, 2008). For example, a stronger climate-related local adaptation has been observed at the range edge of *Fagus sylvatica* (Kreyling et al., 2014). Nevertheless, local adaptation does not guarantee persistence under rapid climatic change. Northern provenances of several boreal conifers show greater potential for performance, whereas southern provenances, which are already near their thermal limits, likely suffer from reduced fitness under further warming (Reich & Oleksyn, 2008; Savolainen, Pyhäjärvi & Knürr, 2007). In general, negative impacts of climate change are often reported at the rear edge of species distributions, where provenances at lower latitudes or elevations may face an increased risk of declines, although this pattern can vary depending on regional and environmental context (Hampe & Petit, 2005; Jump, Hunt & Penuelas, 2006).

Climate projections suggest that the geographic distribution of climatically suitable conditions for many forest tree species may shift toward higher latitudes and elevations under future climate scenarios (Iverson et al., 2008; Penuelas et al., 2007; Rehfeldt et al., 2012). Although natural migration of some tree provenances to such areas has been observed (Alfaro-Ramírez et al., 2017; Lenoir et al., 2008; Trant, Higgs & Starzomski, 2020), the pace has not been sufficient to keep up with the changing climate, thereby placing increasing stress in these provenances. Assisted migration involves the relocation of individuals to anticipate or respond to ongoing environmental changes (Aitken et al., 2008). By guiding tree provenances to areas that are more suitable for their climatic needs (Leech, Almuedo & O’Neill, 2011; Nigh, 2014; Wang et al., 2006), assisted migration can maintain forest health and productivity.

*Betula ermanii* Cham. is a wind-pollinated deciduous tree species distributed across cool-temperate and subalpine regions of Northeast Asia, where it occurs in cold and snowy environments. This species is a major component of deciduous broad-leaved forests, especially in the subalpine region and at the margins of forest distribution, where it forms pure stands before giving way to coniferous forests at higher elevations (Miyawaki, 1984; Okitsu, 1991). Therefore, *B. ermanii* plays a remarkable role in maintaining productivity across mountainous forest regions in Japan. Distinct intraspecific variations in leaf traits have also been reported (Takahashi & Miyajima, 2008). Previous studies reported that growth, morphology, and physiology differ genetically between upper tree-line provenances and other provenances at lower elevations (Wang et al., 2018; Wu et al., 2013; Yu et al., 2014).

In a common garden experiment with 11 seed-source provenances of *B. ermanii*, seedlings from high-altitude origins demonstrated reduced growth performance. Furthermore, significant latitudinal clines were detected in seed weight and key leaf traits, including leaf nitrogen content and specific leaf area (Paing et al., 2021). Building on this, 11 provenance trials were established across Japan, from Hokkaido to Miyazaki Prefecture, in autumn 2019 and spring 2020 (Paing et al., 2023). Subsequent analyses revealed that edge provenances at southern low-altitude and central high elevation showed lower growth than the other provenances (Aihara et al., 2023). The southern rear-edge provenance, Shakaga-Take (SHK_p_), is situated at the highest levels of total precipitation (PRT) for this species, whereas the high-altitude limit provenance, Alps-West (APW_p_), is located at the cold limit of *B. ermanii* (Aihara et al., 2023). These results indicate that *B. ermanii* provenances at distributional margins might be more vulnerable to environmental stress, which is consistent with their positions near climatic limits. However, while previous studies have identified differences in growth performance among provenances, it remains unclear how these differences translate into quantitative responses to climatic mismatches between provenance origin and planting sites. In particular, it is unknown whether provenances from contrasting range-edge positions respond similarly or differently to changes in temperature and precipitation.

Provenance trials and common garden experiments also contribute to *ex situ* conservation as they preserve the genetic material outside the natural range and provide opportunities for studying adaptive variation under controlled conditions (Aitken & Bemmels, 2016; Sáenz-Romero et al., 2020). Thus, such trials can guide *in situ* management and *ex situ* strategies, including the potential use of assisted migration for restoration and conservation under climate change. In this study, statistical models were developed to quantify the effects of climatic transfer distances on the performance of 10 *B. ermanii* provenances planted across multiple sites in Japan. Using temperature- and precipitation-based predictors, we evaluated how climatic mismatches influence seedling performance and how these relationships vary among provenances. The models were further applied to future climate scenarios to project changes in provenance performance and spatial suitability. The following hypotheses were tested: (1) climatic differences between planting site and provenance origin will negatively affect seedling performance; (2) responses to climatic differences will vary among provenances, particularly between provenances originating from range-edge environments; (3) incorporating provenance-specific climatic response will improve predictions of future performance and provide insights relevant to assisted migration strategies.

## Materials & Methods

### Seedling production from provenances

**Figure 1:**
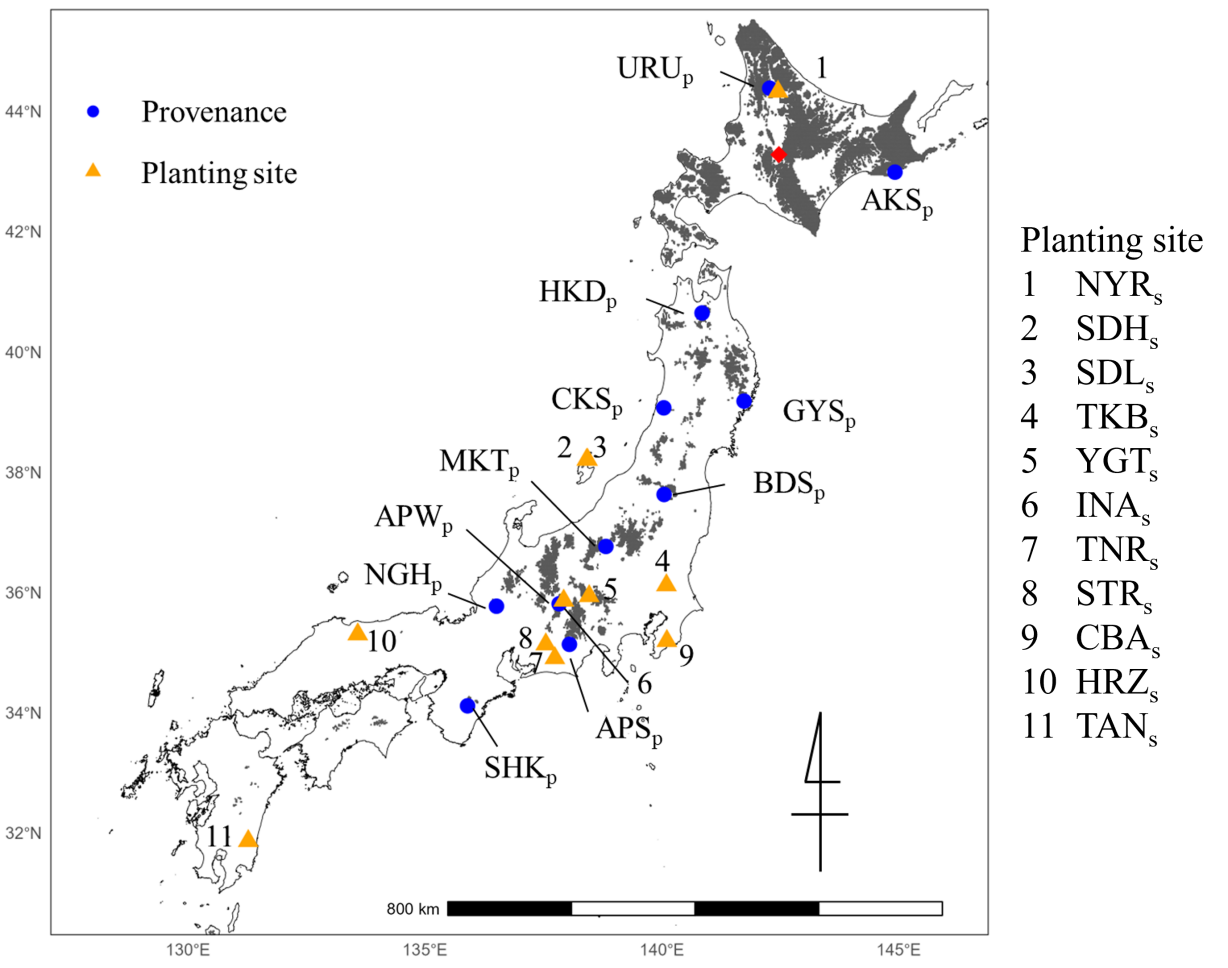
Locations of provenances (blue closed circles) and planting sites (orange closed triangles) throughout Japan. The gray area represents the natural distribution of *B. ermanii*.

Eleven provenance trials scattered throughout Japan were established to evaluate the performance of different provenances of *B. ermanii* undeccccccr varying climatic conditions. “Provenance” refers to the geographic origin of the seed source, whereas “planting site” refers to the experimental site (provenance trial) where seedlings were transplanted and grown. In 2016 and 2017, seeds were collected from 11 provenances of *B. ermanii* growing throughout Japan: Uryu (URU_p_) and Akkeshi (AKS_p_) in Hokkaido; Hakkoda (HKD_p_), Goyo-San (GYS_p_), Choukai-San (CKS_p_), and Bandai-San (BDS_p_) in Tohoku; Mikuni-Touge (MKT_p_), the Alps-West (APW_p_), Nougouhaku-San (NGH_p_), and the Alps-South (APS_p_) in Chubu; and SHK_p_ in the Kinki region (Fig. 1; Table 1).

**Table 1:** Location and climatic variables of planting sites and seed source provenances.

Location	Abb	Lat	Long	Alt	MAT	MDMC	MTWQ	PRT	PWQ	PCQ
Planting site	NYR_s_	44.329	142.454	99	6.4	−12.1	18.4	1,246	454	242
	SDL_s_	38.207	138.418	350	11.3	−1.5	21.6	2,514	726	524
	SDH_s_	38.206	138.435	800	9.8	−3.3	20.4	2,737	792	569
	TKB_s_	36.120	140.100	30	15.1	−1.5	25.0	1,274	444	127
	YGT_s_	35.940	138.470	1,350	8.1	−11.1	18.8	1,661	730	142
	INA_s_	35.865	137.932	770	11.4	−9.9	22.6	1,311	491	138
	HRZ_s_	35.304	133.586	590	11.8	−4.1	22.0	2,804	909	601
	CBA_s_	35.193	140.109	294	15.4	2.3	24.1	2,025	617	358
	STR_s_	35.137	137.554	684	12.3	−4.3	22.3	2,386	865	238
	TNR_s_	34.908	137.746	415	14.1	−1.3	23.9	2,242	786	218
	TAN_s_	31.866	131.275	180	17.0	2.5	25.8	2,692	1,021	239
Provenance	URU_p_	44.386	142.280	487	4.8	−13.6	17.0	2,032	709	418
	AKS_p_	42.990	144.923	105	6.3	−7.8	15.9	1,151	446	140
	HKD_p_	40.648	140.852	898	5.8	−13.6	17.5	2,496	766	657
	GYS_p_	39.182	141.738	842	7.9	−8.1	18.5	2,502	976	256
	BDS_p_	37.629	140.051	1,076	7.3	−9.4	18.5	2,417	896	456
	MKT_p_	36.765	138.823	1,293	6.4	−11.4	17.8	2,119	827	344
	APW_p_	35.811	137.836	2,457	1.5	−19.6	12.6	2,566	950	290
	NGH_p_	35.771	136.513	1,495	6.8	−10.8	17.6	4,035	1,295	876
	APS_p_	35.137	138.053	1,523	7.7	−9.9	17.9	3,434	1,280	327
	SHK_p_	34.114	135.903	1,780	7.4	−8.9	17.5	4,226	1,667	380

**Notes.**

Abb abbreviations of planting sites and provenances (seed source origins) corresponding to Fig. 1 Lat latitude Long longitude Alt altitude MAT mean annual temperature (Bio1) MDMC mean daily minimum temperature of the coldest month (Bio6) MTWQ mean temperature of the warmest quarter (Bio10) PRT Annual precipitation (Bio12) PWQ precipitation during the warmest quarter (Bio18) PCQ precipitation during the coldest quarter (Bio19)

Climatic variables were calculated from the CHELSA database (https://www.chelsa-climate.org/).

Four provenances CKS_p_, APW_p_, NGH_p_, and SHK_p_ originate from the upper montane to subalpine environments near the upper distributional limit of *B. ermanii* on their respective mountains. These sites are characterized by harsh climatic conditions, such as strong winds, heavy snow, and low temperatures (Takahashi, Hirosawa & Morishima, 2012). Notably, APW_p_ is situated at the highest altitude among the provenances, representing the altitudinal limit of *B. ermanii*’s natural distribution, whereas SHK_p_ is located at the southernmost edge, marking the rear edge of its range (Aihara et al., 2023).

The collected seeds were sown in a nursery in April 2018, and by June of the same year, the newly germinated seedlings were transferred into 150-cm^3^ JFA containers. The containerized seedlings were nurtured in a greenhouse for two growing seasons (2018 and 2019). Each seedling, which was derived from different provenances, was tagged with colored vinyl tape and assigned with unique IDs. For most provenances, 20 seedlings, except for GYS_p_, AKS_p_, and CKS_p_, were selected. Consequently, a total of 183 seedlings per site (10 from GYS_p_, nine from AKS_p_, four from CKS_p_, and 20 from each of the other eight provenances) were obtained, yielding a total of 2013 seedlings planted throughout the study (11 sites ×183 seedlings). In August 2019, seedling heights were measured, and the seedlings were delivered for planting (Paing et al., 2021).

### Establishment of provenance trials

In autumn 2019 and spring 2020, containerized seedlings were planted at 11 planting sites across Japan: Nayoro (NYR_s_), Sado High Altitude (SDH_s_), Sado Low Altitude (SDL_s_), Tsukuba (TKB_s_), Yatsugatake (YGT_s_), Ina (INA_s_), Tenryu (TNR_s_), Hiruzen (HRZ_s_), Chiba (CBA_s_), Shitara (STR_s_), and Tano (TAN_s_; Fig. 1, Table 1). Southern planting sites were located outside the natural distribution range of *B. ermanii*. At each site, seedlings were arranged at 1.6-m intervals using a randomized planting design to accommodate micro topographical and environmental differences. After planting, measurements of height and survival counting were conducted for 3 years (Paing et al., 2023).

### Definition and calculation of seedling performance (performance index, PI)

Seedling survival was assessed in autumn 2023, focusing on individuals more than 10 cm high at the time of transplantation. To ensure consistent sample size across analyses, the CKS_p_ provenance was excluded from all analyses due to its very small sample size after establishment, resulting in a total of 1,969 seedlings used as the initial dataset.

To reduce the influence of initial size differences unrelated to climatic conditions, only seedlings above this threshold were included in the analysis. Individuals smaller than 10 cm were excluded because their mortality was likely driven by establishment failure or initial size disadvantage rather than environmental stress. After this filtering step, a total of 1,622 seedlings (out of 1,969 originally planted) were retained for all subsequent analyses.

While the experimental design initially included 179 seedlings per site (after exclusion of CKS_p_), filtering and subsequent mortality resulted in variation in sample size among provenance × site combinations. The number of seedlings retained ranged from 0 to 20, with most combinations including between approximately 8 and 20 individuals. A small number of combinations had very low sample sizes (≤5 individuals), including two cases where no individuals remained after filtering.

Survival rate was calculated for each provenance at each planting site as the proportion of surviving individuals relative to the number of seedlings included in the analysis (*i.e.,* after filtering). Thus, survival estimates represent conditional survival of established seedlings rather than overall establishment success. To integrate growth and survival into a single measure of early performance, a performance index (PI) was calculated following approaches commonly used in transplant and provenance studies (Montalvo & Ellstrand, 2000; Sáenz-Romero et al., 2020). The PI was defined as:

*PI* = *H̀* × *S*

where *H̀* is the mean height of surviving seedlings for a given provenance at a given site, and *S* is the corresponding survival rate. This multiplicative formulation represents the expected growth of established seedlings by weighting mean height by the probability of survival, thereby integrating two interdependent components of early-life performance. Similar indices have been widely used in common garden and transplant experiments as pragmatic measures of relative performance across environments (Ishizuka & Goto, 2012; Savolainen, Pyhäjärvi & Knürr, 2007; Sáenz-Romero et al., 2020). In this study, PI is therefore interpreted as a relative indicator of post-establishment performance, suitable for comparative analyses among provenance × site combinations within a consistent analytical framework, rather than as an absolute measure of lifetime fitness or recruitment success.

### Climatic variables of site and provenance

The climatic differences between each provenance (seed source origin) and its planting site (provenance trial) were quantified to simulate climate change conditions. Climatic data were obtained from high-resolution gridded datasets CHELSA version 2.1 (Karger et al., 2017). CHELSA version 2.1 provides interpolated long-term climate data for the period 1981–2010 at a spatial resolution of 30 arc-seconds (approximately 1 km^2^). The temperature fields are generated using statistical downscaling of reanalysis-based atmospheric data (ERA5), while precipitation is modeled using orographic predictors (*e.g.*, wind fields, valley exposure, and boundary layer height), followed by bias correction procedures. As with other gridded climate products, CHELSA represents spatially continuous estimates of long-term climatic conditions rather than direct measurements at specific locations. CHELSA was selected because it explicitly accounts for topographic effects that are particularly important in mountainous regions such as Japan. All 19 bioclimatic variables were initially extracted, six of which were selected for further analysis, based on their ecological relevance and previous research identifying their significance in explaining the distribution of *B. ermanii* and other cool temperate and subalpine tree species in Japan (Aihara et al., 2023; Shitara et al., 2021; Tsuda et al., 2015; Tsuyama et al., 2020). The selected variables were Bio1, Mean Annual Temperature (MAT); Bio6, Mean Daily Minimum Temperature of the Coldest Month (MDMC); Bio10, Mean Temperature of the Warmest Quarter (MTWQ); Bio12, Annual Precipitation (PRT); Bio18, Precipitation during the Warmest Quarter (PWQ); Bio19, Precipitation during the Coldest Quarter (PCQ).

Then, the climatic differences between planting sites and their corresponding provenance origins were calculated as follows:

ΔMAT= MAT_s_− MAT_p_,

ΔMDMC = MDMC_s_− MDMC_p_,

ΔMTWQ = MTWQ_s_− MTWQ_p_,

ΔPRT = (PRT_s_ − PRT_p_) / PRT_p_,

ΔPWQ = (PWQ_s_ − PWQ_p_) / PWQ_p_,

ΔPCQ = (PCQ_s_ − PCQ_p_) / PCQ_p_,

where the subscript _s_ refers to the planting site and _p_ refers to provenances. The relative differences in precipitation variables were used to account for the variation in baseline precipitation levels across locations, which is consistent with the approach used in previous species distribution modeling studies, such as those conducted for *Abies sachalinensis* (Tsuyama et al., 2020).

### Modeling and validation of climatic transfer effects on seedling performance

To evaluate how climatic differences between provenance origins and planting sites influence seedling performance, generalized linear mixed models (GLMMs) were fitted using the glmmTMB package in R (R Core Team, 2022). The response variable was the performance index (PI), which integrates seedling survival and growth. The PI was modeled at the provenance × planting site level as a continuous response variable. Climatic transfer distances (*i.e.,* differences between planting site and provenance origin) were used as fixed effects. To account for hierarchical structure and unmeasured site-specific variability, planting site ID was included as a random effect , and provenance was initially included as a random effect.

Prior to model fitting, all climatic predictors were mean-centered to improve model interpretability and reduce collinearity between linear and higher-order terms. Quadratic terms were initially included for each climatic variable to allow for potential non-linear (unimodal) relationships between climatic transfer distance and seedling performance, enabling the detection of climatic optima.

Pairwise correlations among climatic variables were evaluated prior to model fitting. Strong correlations were observed between ΔMAT (Bio1) and ΔMDMC (Bio6), and between ΔPRT (Bio12) and ΔPWQ (Bio18), with correlation coefficients exceeding 0.9. To reduce multicollinearity among predictors, only combinations of variables with correlation coefficients <0.7 were retained for model construction.

Model selection was conducted in two steps. In the first step, candidate models were first constructed using either fully linear or fully quadratic formulations, without mixing linear and quadratic terms within the same model. This approach was used to limit the size of the candidate model set and to ensure consistent biological interpretation, as quadratic formulations represent the hypothesis of unimodal responses to climatic transfer. Subsequently, unsupported quadratic terms were removed based on statistical support to obtain a more parsimonious model. Model performance was compared using Akaike Information Criterion (AIC), Bayesian Information Criterion (BIC), log-likelihood, and marginal and conditional *R*^2^ values. The best-supported model included transfer distances in mean temperature of the warmest quarter (ΔMTWQ) and annual precipitation (ΔPRT), with quadratic terms initially included for both variables. However, the quadratic term for ΔMTWQ was not statistically supported (*p* = 0.846) and was therefore removed to improve model parsimony, while the quadratic term for ΔPRT was retained.

In the second step, interaction terms between climatic predictors and provenance were incorporated to evaluate genotype-by-environment (G × E) effects. Based on the refined baseline model, interactions were introduced to test whether provenance-specific responses differed along climatic gradients. Competing models including different combinations of interaction terms were compared using AIC. The final model structure was selected based on model support and parsimony, allowing selected climatic effects to vary among provenances while retaining shared effects where interactions were not supported.

Performance was modeled using a Tweedie distribution with a log link, which is appropriate for non-negative, right-skewed data with potential zero inflation. Model adequacy was evaluated using residual diagnostics, overdispersion checks, and goodness-of-fit metrics implemented in the performance package.

Predictive performance was assessed using five-fold cross-validation. The dataset was randomly partitioned into five subsets; in each iteration, four subsets were used for model training and the remaining subset for testing. Predictive accuracy was quantified using root mean square error (RMSE) and mean absolute error (MAE), and agreement between observed and predicted values was evaluated graphically. For models including quadratic terms, climatic optima were estimated analytically as the vertex of the fitted quadratic function (−β_1_/2β_2_), where β_1_ and β_2_ represent the linear and quadratic coefficients. These optima were back-transformed to the original scale of climatic transfer distance to facilitate ecological interpretation.

### Seedling performance in future climates

Climate projections from WorldClim version 2.1 were used to project the future performance of *B. ermanii* (Hijmans et al., 2005). Although CHELSA data were used for model fitting, future projections were derived from WorldClim because it provides a consistent set of downscaled climate projections across multiple global climate models (GCMs) and emission scenarios. As the two datasets differ in their interpolation and downscaling approaches, the projections should be interpreted as indicative of relative changes in performance rather than exact predictions of absolute values.

Future climate data were acquired for three global climate models (GCMs; MIROC6, HadGEM3-GC31-LL, and MPI-ESM1-2-HR) to account for the inter-model variability for two of the five shared socioeconomic pathways (SSP) of the IPCC Sixth Assessment Report, SSP2–4.5 and SSP5–8.5 scenarios during 2070–2099 (2070s). SSP2–4.5 represents a moderate-warming scenario (a likely increase of 2.1 °C–3.5 °C for ca. 2081–2100), and SSP5–8.5 represents the most extreme warming scenario (a likely increase of 3.3 ° C–5.7 °C for ca. 2081–2100).

Climatic variables under baseline climatic conditions were derived as described in Section Climatic variables of site and provenance. Here, “baseline climatic conditions” refer to long-term averages derived from gridded climate datasets (1981–2010 for CHELSA), rather than direct contemporary measurements. Future projections were obtained from the three GCMs under both SSP scenarios. Climatic transfer distances were recalculated under each future scenario relative to the climatic conditions of each provenance origin, and these values were used to estimate projected performance using the fitted model. Only model that met validation criteria were used for projection.

For *in situ* evaluation, predicted performance values were extracted at the geographic coordinates corresponding to the origin of each provenance, resulting in one value per GCM. The ensemble mean performance was calculated as the arithmetic mean across the three GCMs, and the associated standard deviation was derived from variation among GCM-specific predictions to quantify inter-model uncertainty.

For spatial visualization, raster predictions were generated separately for each GCM and then averaged on a pixel-wise basis to produce ensemble mean maps for each scenario. All spatial analyses and visualizations were conducted using the raster and terra packages in R.

## Results

### Climatic drivers and provenance-specific responses of seedling performance in *B. ermanii*

Seedling performance of *B. ermanii* was associated with climatic transfer distances between planting sites and provenance origins. Climatic transfer distances in mean temperature of the warmest quarter (ΔMTWQ) and annual precipitation (ΔPRT) were the important drivers of variation in performance (Table S1). The final baseline model indicated that seedling performance responded primarily to these two climatic gradients, with a non-linear effect of precipitation initially supported but not retained in subsequent model refinement.

**Figure 2:**
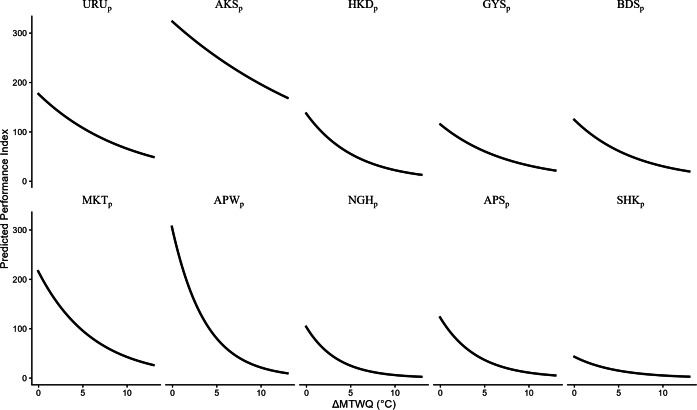
Population-specific responses of seedling performance to mean temperature of the warmest quarter mismatch (ΔMTWQ). Predicted performance index (PI) as a function of mean temperature of the warmest quarter difference between site and provenance (ΔMTWQ), derived from the best-supported GLMM with a Tweedie distribution. Lines represent model predictions for each population, shown in separate panels. Other climatic variables were held constant at their mean values.

In the interaction analysis, allowing the effect of ΔMTWQ to vary among provenances significantly improved model fit (ΔAIC ≈ 10; likelihood ratio test: *χ*^2^ = 43.98, *df* = 17, *p* < 0.001) (Table S2), indicating strong genotype-by-environment (G × E) variation in temperature sensitivity (Fig. 2). In contrast, ΔPRT did not show significant provenance-specific variation. No statistically supported climatic optimum was detected for either temperature or precipitation within the observed range of transfer distances.

**Figure 3:**
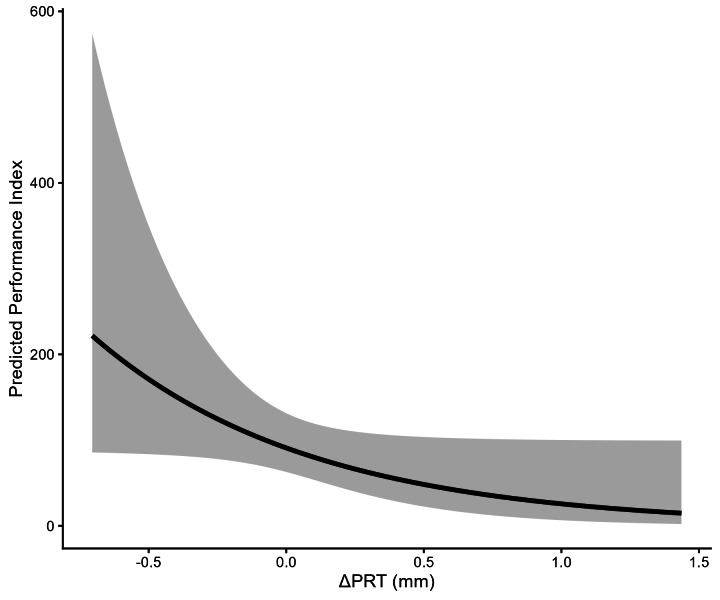
Effect of annual precipitation mismatch (ΔPRT) on seedling performance. Predicted relationship between performance index (PI) and the difference in annual precipitation between planting site and provenance origin (ΔPRT) based on the best-supported GLMM. The solid line represents model predictions, and the shaded area indicates 95% confidence intervals. Other predictors were held at their mean values.

Across provenances, seedling performance tended to decline as annual precipitation at the planting site exceeded that at the provenance origin (ΔPRT; β ≈ −1.26), although this effect was marginally significant (*p* ≈ 0.05, Fig. 3). However, temperature responses varied substantially among provenances. Provenance-specific slopes for ΔMTWQ ranged from weak (*e.g.*, AKS_p_: β ≈ −0.05; URU_p_: β ≈ −0.10) to much stronger negative responses (*e.g.*, NGH_p_: β ≈ −0.28; APW_p_: β ≈ −0.26) (Table 2). This approximately fivefold difference in slope magnitude indicates heterogeneity in sensitivity to temperature mismatch among provenances (Table 2, Fig. 2).

**Figure 4:**
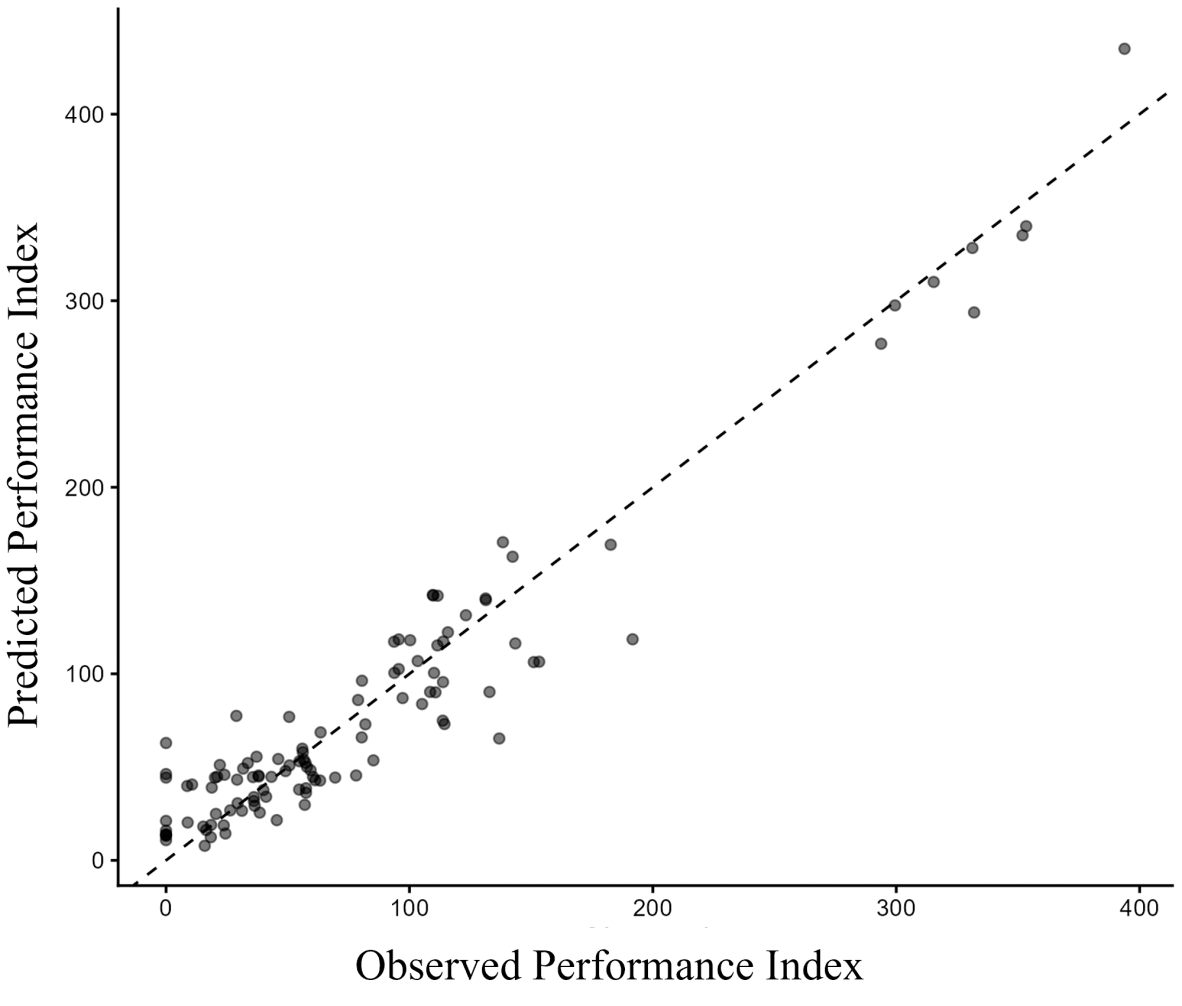
Observed *versus* predicted seedling performance (PI) from the best-supported GLMM. Observed PI is plotted against model-predicted values. The dashed line indicates the 1:1 relationship. Points closer to the line indicate better agreement between observed and predicted values.

Overall, model explained a substantial proportion of the variation in seedling performance (marginal *R*^2^ = 0.577; conditional *R*^2^ = 0.875). Predicted values were positively associated with observed performance (Fig. 4), and cross-validation indicated moderate predictive accuracy (RMSE = 42.96, MAE = 28.80), corresponding to approximately 52% of the mean observed performance (mean = 83.15, SD = 83.71). These results indicate pronounced provenance-specific sensitivity to temperature mismatch, while precipitation effects were weaker and more consistent.

**Table 2:** Estimated parameters of the generalized linear mixed model (GLMM) for seedling performance across provenances.

Provenance	Performance			
	Intercept	ΔMTWQ	ΔPRT	ΔPRT^2^
URU_p_	4.665	−0.099	−1.265	0.135
AKS_p_	5.521	−0.050	−1.265	0.135
HKD_p_	3.985	−0.181	−1.265	0.135
GYS_p_	4.083	−0.128	−1.265	0.135
BDS_p_	4.095	−0.141	−1.265	0.135
MKT_p_	4.536	−0.162	−1.265	0.135
APW_p_	4.342	−0.264	−1.265	0.135
NGH_p_	3.175	−0.283	−1.265	0.135
APS_p_	3.563	−0.240	−1.265	0.135
SHK_p_	2.683	−0.210	−1.265	0.135
Mean	4.065	−0.176	−1.265	0.135

**Notes.**

GLMM generalized linear mixed model ΔMTWQ difference in mean temperature of the warmest quarter between planting site and provenance origin ΔPRT difference in annual precipitation ΔPRT^2^ quadratic term of annual precipitation difference

Performance is defined as mean height × survival rate at the provenance × site level.

### Predicted *in situ* and *ex situ* performance

#### *In situ* performance at provenance origins

The predicted *in situ* performance at provenance origins differed among regions and climate scenarios (Table 3). Under baseline climatic conditions, Hokkaido provenances showed higher predicted performance (*e.g.*, AKS_p_: 277.1), whereas Tohoku provenances exhibited intermediate values (*e.g.*, HKD_p_: 117.2; GYS_p_: 98.7; BDSp: 106.6). Chubu provenances showed a wider range of values (*e.g.*, MKT_p_: 184.3; NGH_p_: 88.3), while the Kinki provenance (SHK_p_) had lower predicted performance (37.1).

Under SSP2–4.5, predicted performance showed variable changes among provenances. Northern provenance (URU_p_: 164.0 ± 21.2) showed increases relative to baseline conditions, whereas most other provenances showed decreases or relatively minor changes. Under SSP5–8.5, predicted performance decreased across all provenances, with larger absolute reductions observed in several Chubu and Kinki provenances (*e.g.*, APW_p_: 60.7 ± 24.4; NGH_p_: 28.6 ± 12.1; SHK_p_: 20.9 ± 6.1).

Across provenance groups, Hokkaido provenances maintained higher predicted performance under all scenarios, whereas Chubu and Kinki provenances generally showed lower values and greater reductions under future scenarios.

### *Ex situ* performance under future climate scenarios

**Figure 5:**
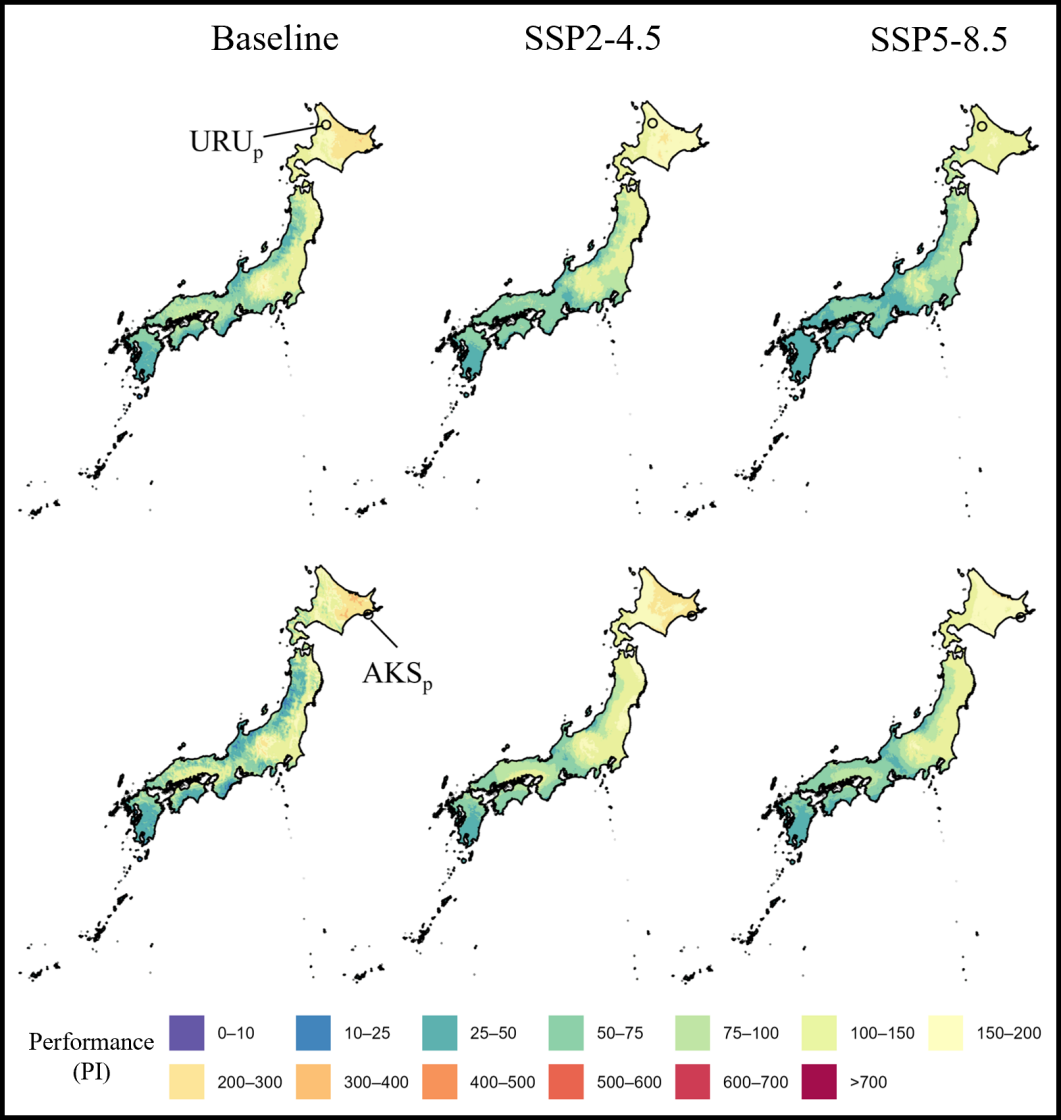
Projected seedling performance (PI) of *Betula ermanii* for Hokkaido provenances across Japan under baseline and future climate scenarios. Panels show spatial distributions of predicted performance for the Hokkaido provenances Uryu (URU_p_) and Akkeshi (AKS_p_) under baseline conditions (1981–2010) and future climate scenarios based on Shared Socioeconomic Pathways (SSP2–4.5 and SSP5–8.5) for the 2070s. Columns represent climate scenarios (baseline, SSP2–4.5, SSP5–8.5), and rows represent provenances (URU_p_ and AKS_p_). Higher predicted performance is generally concentrated in northern regions, particularly within Hokkaido, with declining values toward central and southern Japan. Colors represent predicted performance index (PI) values, and circles indicate provenance origins. Projections are based on ensemble means across multiple global climate models. Administrative boundaries were obtained from the GADM (https://gadm.org/).

**Figure 6:**
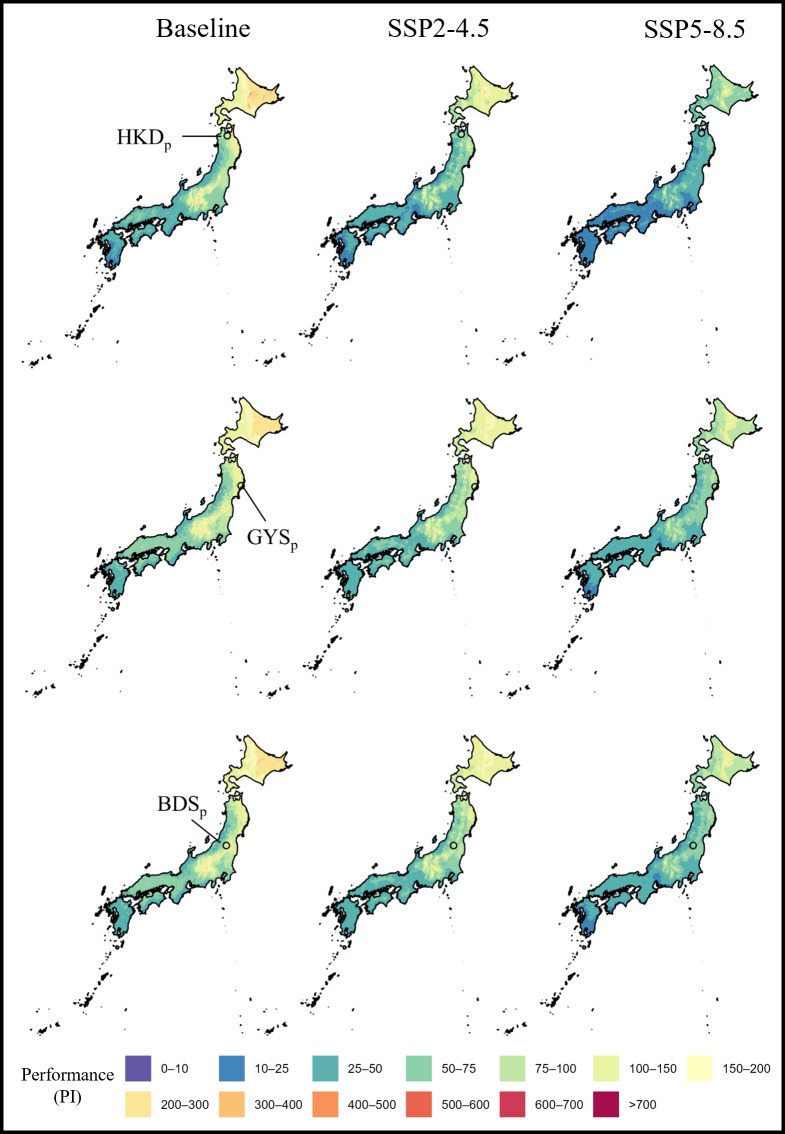
Projected seedling performance (PI) of *Betula ermanii* for Tohoku provenances across Japan under baseline and future climate scenarios. Panels show spatial distributions of predicted performance for the Tohoku provenances Hakkoda (HKD_p_), Goyo-San (GYS_p_), and Bandai-San (BDS_p_) under baseline conditions (1981–2010) and future climate scenarios based on Shared Socioeconomic Pathways (SSP2–4.5 and SSP5–8.5) for the 2070s. Columns represent climate scenarios (baseline, SSP2–4.5, SSP5–8.5), and rows represent provenances. A north–south gradient is evident, with higher predicted performance concentrated in Hokkaido and decreasing toward central and southern Japan, although areas of higher performance are more restricted compared to Hokkaido provenances. Colors represent predicted performance index (PI) values, and circles indicate provenance origins. Projections are based on ensemble means across multiple global climate models. Administrative boundaries were obtained from the GADM (https://gadm.org/).

**Figure 7:**
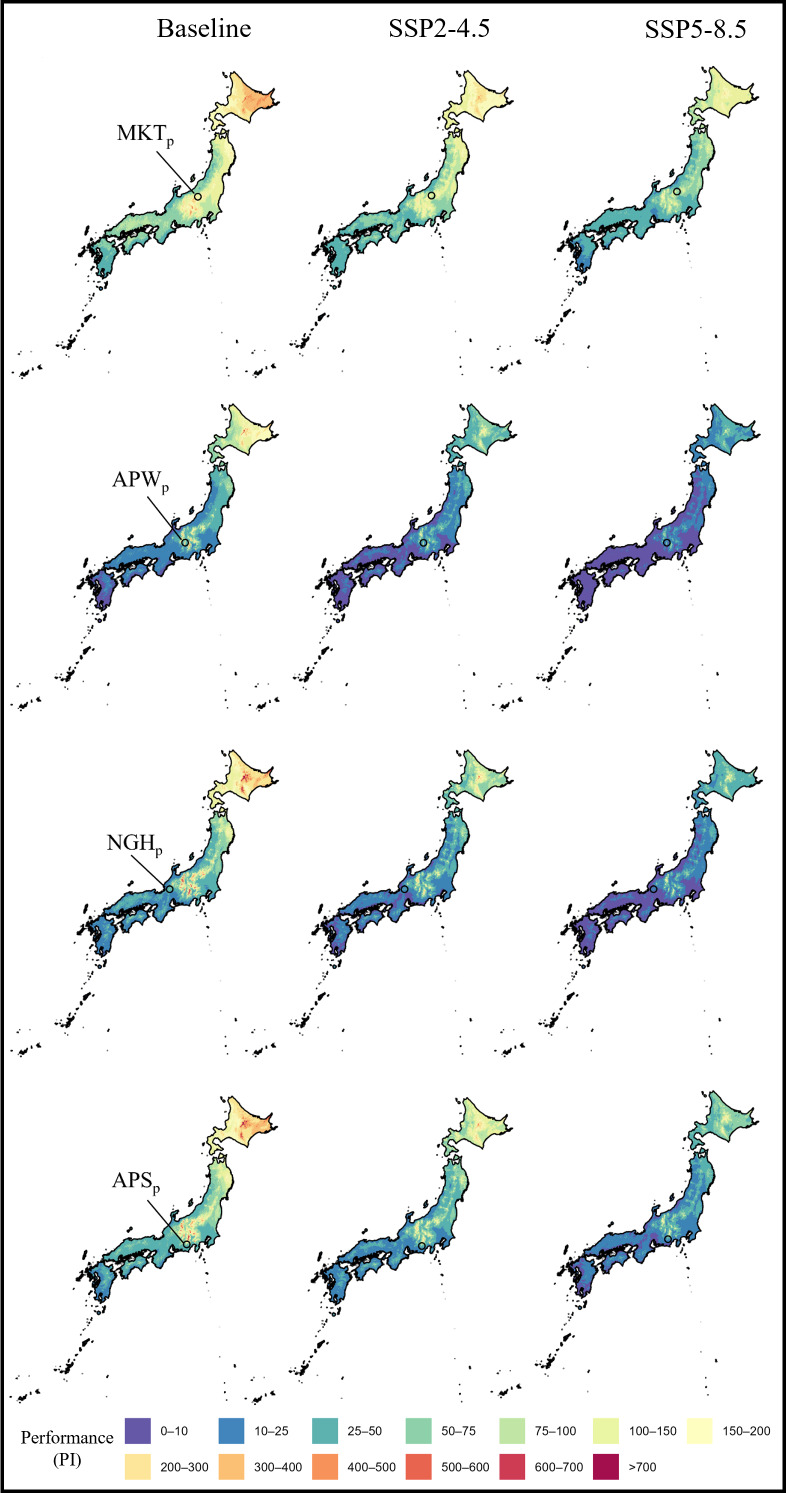
Projected seedling performance (PI) of *Betula ermanii* for Chubu provenances across Japan under baseline and future climate scenarios. Panels show spatial distributions of predicted performance for the Chubu provenances Mikuni-Touge (MKT_p_), Alps-West (APW_p_), Nougouhaku-San (NGH_p_), and Alps-South (APS_p_) under baseline conditions (1981–2010) and future climate scenarios based on Shared Socioeconomic Pathways (SSP2–4.5 and SSP5–8.5) for the 2070s. Columns represent climate scenarios (baseline, SSP2–4.5, SSP5–8.5), and rows represent provenances. Predicted performance shows greater spatial heterogeneity compared to northern provenances, with moderate values distributed across central and eastern Japan and fewer areas of high performance. Colors represent predicted performance index (PI) values, and circles indicate provenance origins. Projections are based on ensemble means across multiple global climate models. Administrative boundaries were obtained from the GADM (https://gadm.org/).

**Figure 8:**
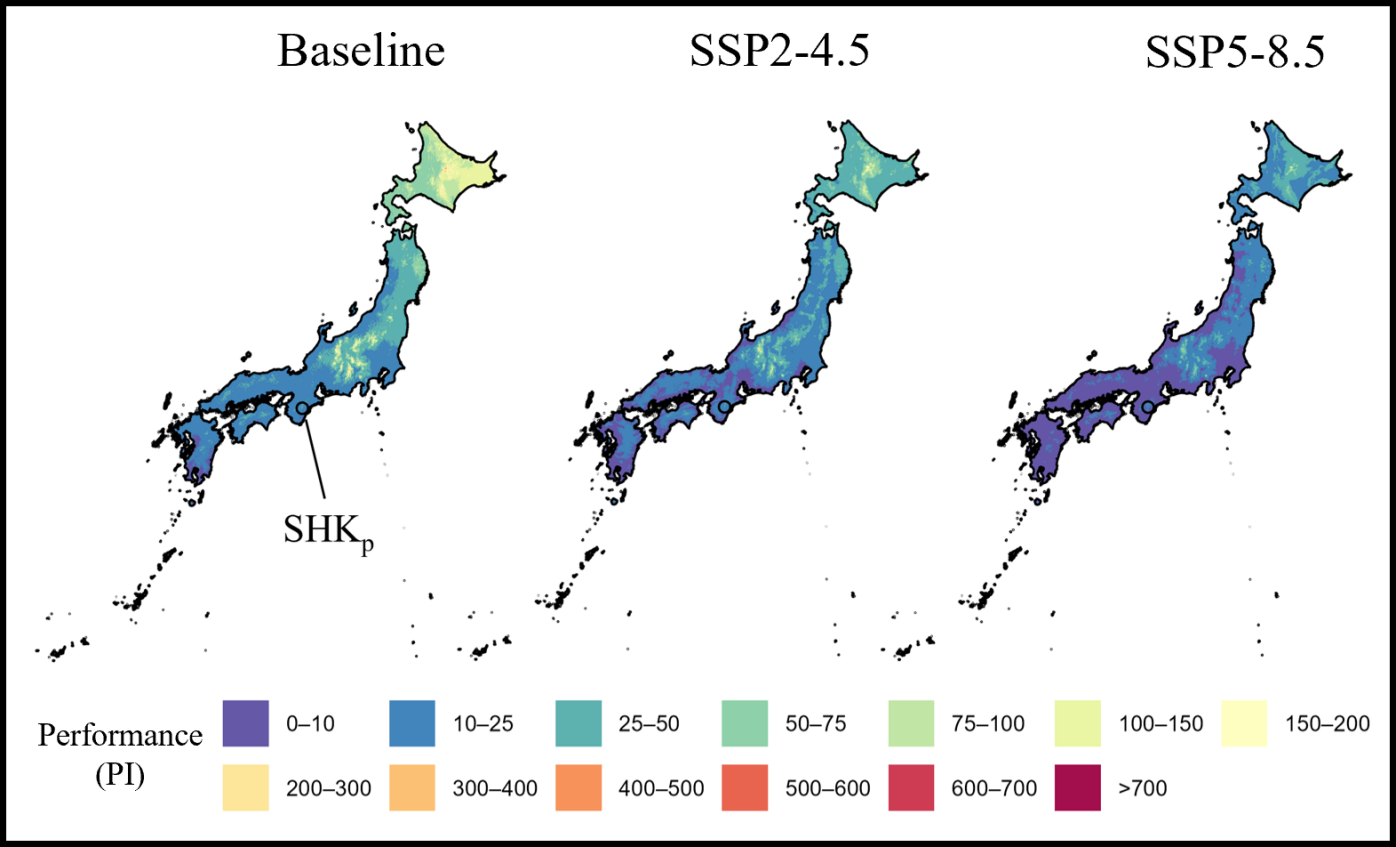
Projected seedling performance (PI) of *Betula ermanii* for the Kinki provenance across Japan under baseline and future climate scenarios. Panels show spatial distributions of predicted performance for the Kinki provenance SHK_p_ under baseline conditions (1981–2010) and future climate scenarios based on Shared Socioeconomic Pathways (SSP2–4.5 and SSP5–8.5) for the 2070s. Columns represent climate scenarios (baseline, SSP2–4.5, SSP5–8.5). Predicted performance is generally low across most regions, with only limited areas of moderate values. Colors represent predicted performance index (PI) values, and circles indicate provenance origins. Projections are based on ensemble means across multiple global climate models. Administrative boundaries were obtained from the GADM (https://gadm.org/).

The projected maps showed spatial patterns in the *ex situ* performance of *B. ermanii* provenances across Japan (Figs. 5–8). Across provenance groups, higher predicted performance values were generally observed in northern regions, particularly in Hokkaido, whereas lower values were more common in central and southern regions.

Spatial patterns differed among provenance groups. Hokkaido provenances showed higher predicted performance across much of Hokkaido, with lower values extending toward central and southern Japan (Fig. 5). Provenances from the Tohoku region showed a similar north–south gradient, although areas with higher predicted performance were more restricted within Hokkaido (Fig. 6). Chubu provenances showed more spatial heterogeneity, with moderate values distributed across parts of central and eastern Japan and fewer areas with higher predicted performance (Fig. 7). The Kinki provenance showed lower predicted performance across most regions, with only limited areas of moderate values (Fig. 8).

Across climate scenarios, the overall spatial structure remained similar. Under SSP2–4.5 and SSP5–8.5, predicted performance values generally decreased relative to baseline conditions. The extent of areas with higher predicted performance was reduced under SSP5–8.5 compared to SSP2–4.5. In addition, the spatial distribution of predicted performance within Hokkaido showed minor variation across scenarios.

**Table 3:** Predicted *in situ* performance of *B. ermanii* provenances under baseline and future climate scenarios.

Provenance	Baseline	SSP2-4.5	SSP5-8.5
URU_p_	151.4	164.0 ± 21.2	124.0 ± 23.9
AKS_p_	277.1	222.7 ± 16.5	183.5 ± 20.1
HKD_p_	117.2	103.2 ± 22.1	66.1 ± 19.4
GYS_p_	98.7	106.8 ± 14.8	77.5 ± 14.3
BDS_p_	106.6	100.5 ± 16.7	72.5 ± 15.7
MKT_p_	184.3	123.7 ± 22.2	86.6 ± 21.3
APW_p_	257.8	106.7 ± 28.7	60.7 ± 24.4
NGH_p_	88.3	52.3 ± 16.6	28.6 ± 12.1
APS_p_	104.5	65.8 ± 15.7	40.2 ± 13.3
SHK_p_	37.1	32.4 ± 6.1	20.9 ± 6.1

**Notes.**

The values refer to the predicted performance at each provenance origin. Predictions were obtained from the GLMM using climatic variables as fixed effects and averaged across three global climate models (GCMs). Baseline: predicted *in situ* performance under the baseline climate (1981–2010); SSP2-4.5: predicted *in situ* performance under SSP2-4.5 scenario across three GCMs (2070s); SSP5-8.5: predicted *in situ* performance under SSP5-8.5 scenario across three GCMs (2070s).

## Discussion

Our analysis of *B. ermanii* Cham. seedling performance across climatic gradients demonstrates that climatic mismatches between provenance origin and planting site are important predictors of early performance, with substantial variation among provenances. By integrating growth and survival, our results provide a robust assessment of post-establishment performance and show that temperature mismatches, particularly through provenance-specific responses, are the primary drivers of variation, while precipitation showed a weaker and only marginally supported association. Consistent with our first hypothesis, performance generally declined with increasing climatic mismatch, particularly when precipitation at the planting site exceeded that of the provenance origin (ΔPRT). This negative relationship suggests that wetter-then-origin conditions may impose physiological stress, potentially through waterlogging, reduced soil oxygen availability, and impaired root function, which collectively limit growth and survival (Ejankowski, 2008; Fernández et al., 1997; Kozlowski, 1997; Schmull & Thomas, 2000; Tang & Kozlowski, 1983; Tang & Kozlowski, 1982; Wang et al., 2017; Wang et al., 2016).

Notably, the responses to climatic transfer were largely linear rather than unimodal, indicating that, within the observed range, provenances are already operating along a gradient of increasing maladaptation rather than near climatic optima. Similar monotonic declines with increasing transfer distance have been reported in provenance trials of forest trees (Rehfeldt et al., 2002; Wang et al., 2006), suggesting that even moderate climatic shifts can lead to measurable reductions in performance. In contrast, mean temperature of the warmest quarter (ΔMTWQ) showed no overall effect when averaged across provenances, but this masked substantial variation among provenances. This suggests that provenance-level responses can be obscured when analyses are conducted at the species level, emphasizing the importance of incorporating intraspecific variation ((Leites & Benito Garzón, 2023; Savolainen, Pyhäjärvi & Knürr, 2007).

A major finding of this study is the strong heterogeneity in temperature sensitivity among provenances, with up to fivefold differences in response slopes. High-elevation central provenances (*e.g.*, APW_p_ and NGH_p_) showed steep declines under warming, whereas northern Hokkaido provenances (URU_p_, AKS_p_) were comparatively insensitive, suggesting broader climatic tolerance or greater plasticity in northern provenances. In contrast, central and high-elevation provenances were more sensitive to temperature increases, indicating potential vulnerability under future warming.

These patterns are consistent with a mechanistic framework in which reduced performance at range edges arises from distinct processes, namely adaptive specialization and genetic load in *B. ermanii* (Aihara et al., 2023). Our results support and extend this interpretation. For high-elevation provenances such as APW_p_ and NGH_p_, the strong negative response to warming likely reflects adaptive specialization to cold, harsh environments, where traits such as reduced growth or delayed phenology are advantageous under short growing seasons but become maladaptive under warmer conditions (Körner, 2021). This interpretation is consistent with findings that reduced size but not growth rate in APW_p_ can be attributed to adaptive selection rather than genetic constraints (Aihara et al., 2023).

In contrast, the consistently low performance of the southern rear-edge provenance SHK_p_ across climatic gradients and scenarios suggests a different mechanism. Rather than showing strong climatic sensitivity, SHK_p_ exhibited intrinsically low performance, which is consistent with the hypothesis of genetic load accumulation and inbreeding depression in small, isolated provenances (Hampe & Petit, 2005; Leroy et al., 2018). This interpretation closely matches genetic evidence indicating reduced diversity and elevated relatedness in this provenance (Aihara et al., 2023), reinforcing that rear-edge vulnerability may be driven more by demographic and genetic factors than by climatic mismatch alone. Together, these findings indicate that similar patterns of reduced performance at range margins can arise from fundamentally different underlying mechanisms, which must be considered when predicting responses to climate change.

While some broad-leaved species, such as *Quercus robur*, show concave performance responses to warming, *B. ermanii* does not appear to experience significant gains under moderate temperature increases (Bresson et al., 2011; De Frenne et al., 2013). This lack of concave response in *B. ermanii* may indicate that the physiological mechanisms of this species are not highly adaptive to gradual warming alone, or that the observed linear response may obscure more complex interactions. In species with low phenotypic plasticity, increases in temperature often do not result in substantial shifts in growth or survival (Nicotra et al., 2010; Valladares, Gianoli & Gómez, 2007). Climate change entails more than just temperature increases; it involves shifts in seasonal patterns, temperature extremes, and changes in precipitation regimes, all of which play vital roles in determining species responses (Allan et al., 2021). For instance, the increasing frequency and intensity of extreme heat events can exacerbate water stress, reduce growth, and lead to mortality, regardless of mean temperature trends (Teskey et al., 2015). Similarly, shifts in seasonal temperature patterns such as earlier spring warming or delayed autumn cooling can disrupt key phenological processes like budburst, flowering, and leaf senescence, with implications for survival and productivity (Richardson et al., 2013; Vitasse, Lenz & Körner, 2014).

Projections under future climate scenarios indicate widespread declines in performance, particularly under SSP5–8.5, supporting the expectation that climate change will negatively affect *B. ermanii* across much of its range. However, the magnitude of decline varied among provenances. Northern provenances (Hokkaido and parts of Tohoku) maintained relatively high performance both *in situ* and across projected spatial distributions, suggesting that Hokkaido may function as a climatic refugium. The persistence of high-performance areas in northern regions is consistent with projected poleward shifts in suitable habitat for cool-temperate and boreal tree species (Iverson et al., 2008; Rehfeldt et al., 2012). At the same time, the overall spatial structure of suitability remained relatively stable across scenarios, indicating that relative differences among regions are robust, even as absolute performance declines.

The strong provenance-specific responses observed in this study provide important guidance for assisted migration strategies. First, northern provenances (*e.g.*, URU_p_, AKS_p_) showed high performance and low sensitivity to warming, making them suitable candidates for assisted gene flow into future climates, particularly within northern Japan. This convergence of present-day and future suitability indicates that Hokkaido could serve as an *in situ* stronghold and a recipient habitat in assisted gene flow strategies (Aitken & Whitlock, 2013; Prober et al., 2015). Such areas may serve as climate refugia that preserve persistence and ecosystem productivity under warming conditions (Chen et al., 2025; Hampe & Petit, 2005; Keppel et al., 2012; Kim et al., 2025). Therefore, preserving such areas is critical for extant provenances, and these areas serve as potential seed/genotype sources to support adaptive ecosystem functioning into the future.

Second, central Chubu provenances (MKT_p_, APW_p_, NGH_p_, and APS_p_) displayed variable responses. In particular, high-elevation provenances such as APW_p_ and NGH_p_ were vulnerable to warming, whereas low-altitude provenances such as MKT_p_ and APS_p_ showed intermediate performance. Nonetheless, their improved performance when projected into northern regions suggests potential for northward assisted migration, or *ex situ* relocation trials for these provenances. However, any such program should proceed with experimental staging, including reciprocal trials and demographic monitoring, to mitigate the risks of maladaptation (Aitken & Bemmels, 2016).

Third, the rear-edge provenance SHK_p_ should not be prioritized for relocation despite its distinct genetic identity. Genetic evidence further identifies SHK_p_ as a diploid relict lineage at the southern range margin of the species, which is distinct from the widespread tetraploid provenances (Aihara, Araki & Tsumura, 2024), emphasizing its evolutionary distinctiveness despite poor ecological performance. Consequently, conservation strategies should focus on safeguarding its genetic resources through seed banking and *in situ* protection rather than expecting high performance under northward relocation. This strategy is consistent with rear-edge conservation frameworks (Hampe & Jump, 2011; Hampe & Petit, 2005), which prioritize preserving unique genetic diversity and evolutionary potential over ecological performance alone and cautions about the risks of shifting locally adapted genotypes (Aitken & Whitlock, 2013). Overall, our results highlight that effective climate adaptation strategies require integrating climatic suitability with provenance-specific traits and genetic context.

Nevertheless, our climate-centric projections have notable limitations. Transplant stress, soil, microbial mismatches, and unmodeled biotic interactions may substantially influence the success of establishing provenance trials (Cuesta et al., 2010). Moreover, our models focus on early survival and growth, not long-term fecundity or stand-level resilience, which are phenomena profoundly influenced by local pests, diseases, and phenological timing (Augspurger, 2013). Notably, genetic considerations such as unique alleles or local adaptations in rear-edge provenances such as SHK_p_ may fall outside pure ecological fitness metrics and warrant preservation regardless of their modeling outcome (Hampe & Petit, 2005). Finally, uncertainties in GCM scenarios and climate projections should temper rapid operationalization; instead, adaptive field trials across multiple sites and seasons are recommended (McKenney et al., 2007).

Our study demonstrates that conservation strategies for *B. ermanii* must be provenance-specific and trait-informed. The northern provenances from Hokkaido and Tohoku deserve *in situ* emphasis, and they can serve as climate-safe donors. Central provenances are promising candidates for assisted migration to central Hokkaido under projected futures. On the contrary, rear-edge provenance should be treated as a genetically unique but ecologically vulnerable lineage requiring targeted genetic safeguarding. Our work provides a quantitative foundation for operationalizing climate-adjusted provenancing, with the caveat that ecological complexity and uncertainty call for staged, monitoring-driven implementation. Moreover, our results support the principle that tree species with wide geographic distributions exhibit substantial intra-specific differentiation and that leveraging provenance-specific knowledge is essential for anticipating climate responses and guiding forest management in a rapidly changing world.

## Conclusions

This study demonstrates that climatic transfer effects on *B. ermanii* are strongly provenance-dependent, with precipitation exerting an overall negative effect and temperature responses varying widely among provenances. By linking these patterns to mechanisms of local adaptation and genetic load, our results extend previous findings and provide a predictive framework for assessing climate change impacts. Our findings emphasize that intraspecific variation is critical for understanding and managing forest responses to climate change. Incorporating provenance-specific climatic responses into conservation planning can improve assisted migration strategies, identify climate refugia, and support adaptive forest management in a rapidly changing environment.

Northern provenances from Hokkaido and Tohoku exhibited higher resilience under projected climates, whereas central Chubu provenances showed reduced performance *in situ* but higher performance when projected northward, supporting the potential role of assisted migration. In contrast, the southern rear-edge provenance represents a genetically distinct but ecologically vulnerable lineage that warrants conservation focused on genetic preservation. Although our projections are based primarily on climate-driven models and early-life performance, they provide a quantitative framework for climate-adjusted provenancing. Future work should integrate long-term field trials, biotic interactions, and demographic processes to better evaluate the effectiveness and risks of assisted migration under ongoing climate change. More broadly, our findings emphasize that accounting for provenance-specific climatic adaptation will be essential for developing resilient forest management strategies in a rapidly warming world.

## Supplemental Information

10.7717/peerj.21425/supp-1 Supplemental Information 1 Model selection results for GLMMs testing the effects of climatic differences (Δ= site − provenance) on seedling performance (PI) Models include fixed effects of climate variables and random effects of provenance and site. Model fit is evaluated using AIC, BIC, logLik, marginal *R*^2^, and conditional *R*^2^. ΔAIC indicates the difference from the best model. Continuous predictors were mean-centered prior to analysis, and quadratic terms were included to account for non-linear relationships. AIC: Akaike Information Criterion, lower values indicate better model fit. BIC: Bayesian Information Criterion penalizes model complexity. logLik: log-likelihood of the model. *R*^2^_marginal_: variance explained by fixed effects. *R*^2^_conditional_: variance explained by fixed and random effects. ΔAIC: Difference in AIC relative to the best model. pop: provenance as random effect. site: planting site as random effect.

10.7717/peerj.21425/supp-2 Supplemental Information 2 Model selection results for GLMMs testing the effects of climatic differences (Δ= site − provenance) × provenance interaction effects on seedling performance (PI) Interaction models include provenance (pop) × climate terms and random effects of site, allowing for provenance-specific climatic responses. AIC: Akaike Information Criterion, lower values indicate better model fit. BIC: Bayesian Information Criterion penalizes model complexity. logLik: log-likelihood of the model. *R*^2^_marginal: variance explained by fixed effects. *R*^2^_conditional: variance explained by fixed and random effects. ΔAIC: Difference in AIC relative to the best model. site: planting site as random effect.

## Additional Information and Declarations

### Data Availability

The following information was supplied regarding data availability: The data is available at Dryad: Paing, Aye Myat Myat (2025). *Betula ermanii* Cham. provenance trial in Japan: Survival, height, diameter, and productivity data (Dataset). Dryad. https://doi.org/10.5061/dryad.2ngf1vhxb.
