# Supplementary material for: Provenance-specific responses to climatic mismatch in Betula ermanii Cham. and implications for climate adaptation
Source: PeerJ. 2026 Jun 11;14:e21425. doi: 10.7717/peerj.21425 (PMC13264976; doi:10.7717/peerj.21425)
Supplement: Supplemental Information 1 — Models include fixed effects of climate variables and random effects of provenance and site. Model fit is evaluated using AIC, BIC, logLik, marginal R2, and conditional R2. ΔAIC indicates the difference from the best model. Continuous predictors were mean-centered prior to analysis, and quadratic terms were included to account for non-linear relationships. AIC: Akaike Information Criterion, lower values indicate better model fit. BIC: Bayesian Information Criterion penalizes model complexity. logLik: log-likelihood of the model. R2marginal: variance explained by fixed effects. R2conditional: variance explained by fixed and random effects. ΔAIC: Difference in AIC relative to the best model. pop: provenance as random effect. site: planting site as random effect. [file peerj-14-21425-s001.docx]

**Table S1.** Model selection results for GLMMs testing the effects of climatic differences (Δ = site − provenance) on seedling performance (PI). Models include fixed effects of climate variables and random effects of provenance and site. Model fit is evaluated using AIC, BIC, logLik, marginal R², and conditional R². ΔAIC indicates the difference from the best model. Continuous predictors were mean-centered prior to analysis, and quadratic terms were included to account for non-linear relationships.

| Model | Fixed effects | Random effects | AIC | BIC | logLik | R²_ marginal | R²_ conditional | ΔAIC |
| --- | --- | --- | --- | --- | --- | --- | --- | --- |
| resp1 | ΔMTWQ + ΔPRT + ΔMTWQ^2^ + ΔPRT^2^ | pop, site | 1059.844 | 1084.148 | -520.922 | 0.321 | 0.777 | 0.000 |
| resp2 | ΔMTWQ + ΔPWQ +ΔMTWQ^2^ + ΔPWQ^2^ | pop, site | 1061.443 | 1085.747 | -521.721 | 0.312 | 0.783 | 1.599 |
| resp3 | ΔMTWQ + ΔPWQ | pop, site | 1063.399 | 1082.302 | -524.699 | 0.266 | 0.720 | 3.555 |
| resp4 | ΔMAT + ΔPRT + ΔMAT^2^ + ΔPRT^2^ | pop, site | 1063.544 | 1087.848 | -522.772 | 0.302 | 0.765 | 3.700 |
| resp5 | ΔMTWQ + ΔPRT | pop, site | 1063.845 | 1082.748 | -524.923 | 0.263 | 0.709 | 4.001 |
| resp6 | ΔMAT + ΔPWQ + ΔMAT^2^ + ΔPWQ^2^ | pop, site | 1063.965 | 1088.270 | -522.983 | 0.299 | 0.734 | 4.121 |
| resp7 | ΔMAT + ΔPCQ | pop, site | 1064.194 | 1083.097 | -525.097 | 0.276 | 0.783 | 4.350 |
| resp8 | ΔMTWQ + ΔPCQ | pop, site | 1064.337 | 1083.240 | -525.168 | 0.253 | 0.776 | 4.493 |
| resp9 | ΔMTWQ + ΔPWQ + ΔPCQ + ΔMTWQ^2^ + ΔPWQ^2^ + ΔPCQ^2^ | pop, site | 1064.661 | 1094.367 | -521.331 | 0.317 | 0.786 | 4.818 |
| resp10 | ΔMDMC + ΔPRT + ΔMDMC^2^ + ΔPRT^2^ | pop, site | 1064.695 | 1088.999 | -523.348 | 0.196 | 0.764 | 4.851 |
| resp11 | ΔMAT + ΔPWQ | pop, site | 1064.877 | 1083.781 | -525.439 | 0.267 | 0.761 | 5.033 |
| resp12 | ΔMTWQ + ΔPWQ + ΔPCQ | pop, site | 1064.896 | 1086.500 | -524.448 | 0.258 | 0.724 | 5.052 |
| resp13 | ΔMAT + ΔPRT | pop, site | 1064.937 | 1083.840 | -525.468 | 0.270 | 0.766 | 5.093 |
| resp14 | ΔMAT + ΔPWQ + ΔPCQ | pop, site | 1065.628 | 1087.232 | -524.814 | 0.265 | 0.764 | 5.784 |
| resp15 | ΔMDMC + ΔPWQ + ΔMDMC^2^ + ΔPWQ^2^ | pop, site | 1065.736 | 1090.040 | -523.868 | 0.200 | 0.766 | 5.892 |
| resp16 | ΔMAT + ΔPWQ + ΔPCQ + ΔMAT^2^ + ΔPWQ^2^ + ΔPCQ^2^ | pop, site | 1065.975 | 1095.681 | -521.988 | 0.316 | 0.783 | 6.131 |
| resp17 | ΔMDMC + ΔPCQ | pop, site | 1066.519 | 1085.422 | -526.259 | 0.180 | 0.784 | 6.675 |
| resp18 | ΔMDMC + ΔPRT | pop, site | 1067.233 | 1086.137 | -526.617 | 0.171 | 0.769 | 7.389 |
| resp19 | ΔMDMC + ΔPWQ | pop, site | 1067.236 | 1086.140 | -526.618 | 0.170 | 0.766 | 7.392 |
| resp20 | ΔMTWQ + ΔPCQ + ΔMTWQ^2^ + ΔPCQ^2^ | pop, site | 1067.365 | 1091.670 | -524.683 | 0.270 | 0.769 | 7.521 |
| resp21 | ΔMAT + ΔPCQ + ΔMAT^2^ + ΔPCQ^2^ | pop, site | 1067.550 | 1091.854 | -524.775 | 0.285 | 0.778 | 7.706 |
| resp22 | ΔMDMC + ΔPWQ + ΔPCQ + ΔMDMC^2^ + ΔPWQ2 + ΔPCQ^2^ | pop, site | 1067.572 | 1097.277 | -522.786 | 0.226 | 0.792 | 7.728 |
| resp23 | ΔMDMC + ΔPWQ + ΔPCQ | pop, site | 1068.187 | 1089.791 | -526.094 | 0.173 | 0.777 | 8.343 |
| resp24 | ΔMDMC + ΔPCQ + ΔMDMC^2^ +ΔPCQ^2^ | pop, site | 1068.896 | 1093.201 | -525.448 | 0.194 | 0.778 | 9.053 |
| resp25 | ΔPWQ + ΔPCQ | pop, site | 1071.114 | 1090.017 | -528.557 | 0.009 | 0.789 | 11.270 |
| resp26 | ΔPWQ + ΔPCQ + ΔPWQ^2^ + ΔPCQ^2^ | pop, site | 1073.272 | 1097.576 | -527.636 | 0.043 | 0.795 | 13.428 |

AIC: Akaike Information Criterion, lower values indicate better model fit. BIC: Bayesian Information Criterion penalizes model complexity. logLik: log-likelihood of the model. R²_marginal: variance explained by fixed effects. R²_conditional: variance explained by fixed and random effects. ΔAIC: Difference in AIC relative to the best model. pop: provenance as random effect. site: planting site as random effect.
