# Supplementary material for: Provenance-specific responses to climatic mismatch in Betula ermanii Cham. and implications for climate adaptation
Source: PeerJ. 2026 Jun 11;14:e21425. doi: 10.7717/peerj.21425 (PMC13264976; doi:10.7717/peerj.21425)
Supplement: Supplemental Information 2 — Interaction models include provenance (pop) × climate terms and random effects of site, allowing for provenance-specific climatic responses. AIC: Akaike Information Criterion, lower values indicate better model fit. BIC: Bayesian Information Criterion penalizes model complexity. logLik: log-likelihood of the model. R2_marginal: variance explained by fixed effects. R2_conditional: variance explained by fixed and random effects. ΔAIC: Difference in AIC relative to the best model. site: planting site as random effect. [file peerj-14-21425-s002.docx]

**Table S2**. Model selection results for GLMMs testing the effects of climatic differences (Δ = site − provenance) × provenance interaction effects on seedling performance (PI). Interaction models include provenance (pop) × climate terms and random effects of site, allowing for provenance-specific climatic responses.

| Model | Fixed effects | Random effects | AIC | BIC | logLik | R²_ marginal | R²_ conditional | ΔAIC |
| --- | --- | --- | --- | --- | --- | --- | --- | --- |
| respop0 | ΔMTWQ + ΔPRT + ΔPRT2 | site | 1057.888 | 1079.492 | -520.944 | 0.312 | 0.750 | 9.983 |
| respop1 | ΔMTWQ*pop + ΔPRT*pop + ΔPRT2*pop | site | 1058.499 | 1174.619 | -486.249 | 0.601 | 0.876 | 10.594 |
| respop2 | ΔMTWQ + ΔPRT*pop + ΔPRT2*pop | site | 1071.432 | 1163.248 | -501.716 | 0.511 | 0.840 | 23.527 |
| respop3 | ΔMTWQ*pop + ΔPRT + ΔPRT2 | site | 1047.905 | 1115.417 | -498.953 | 0.577 | 0.875 | 0.000 |

AIC: Akaike Information Criterion, lower values indicate better model fit. BIC: Bayesian Information Criterion penalizes model complexity. logLik: log-likelihood of the model. R²_marginal: variance explained by fixed effects. R²_conditional: variance explained by fixed and random effects. ΔAIC: Difference in AIC relative to the best model. site: planting site as random effect.
